# Nanoscale‐tipped wire array injections transfer DNA directly into brain cells *ex vivo* and *in vivo*

**DOI:** 10.1002/2211-5463.13377

**Published:** 2022-03-15

**Authors:** Rika Numano, Akihiro Goryu, Yoshihiro Kubota, Hirohito Sawahata, Shota Yamagiwa, Minako Matsuo, Tadahiro Iimura, Hajime Tei, Makoto Ishida, Takeshi Kawano

**Affiliations:** ^1^ 13129 Department of Applied Chemistry and Life Science Toyohashi University of Technology Japan; ^2^ 13129 Electronics‐Inspired Interdisciplinary Research Institute (EIIRIS) Toyohashi University of Technology Japan; ^3^ 13129 Department of Electrical and Electronic Information Engineering Toyohashi University of Technology Japan; ^4^ 12820 National Institute of Technology Ibaraki College Japan; ^5^ 12810 Department of Pharmacology Graduate School of Dental Medicine Hokkaido University Sapporo Japan; ^6^ 12858 Graduate School of Natural Science and Technology Kanazawa University Japan

**Keywords:** circadian rhythms, clock genes, *ex vivo*, nanoscale‐tipped wire, NTW array injection, short hairpin RNA

## Abstract

Genetic modification to restore cell functions in the brain can be performed through the delivery of biomolecules in a minimally invasive manner into live neuronal cells within brain tissues. However, conventional nanoscale needles are too short (lengths of ~10 µm) to target neuronal cells in ~1‐mm‐thick brain tissues because the neuronal cells are located deep within the tissue. Here, we report the use of nanoscale‐tipped wire (NTW) arrays with diameters < 100 nm and wire lengths of ~200 µm to address biomolecule delivery issues. The NTW arrays were manufactured by growth of silicon microwire arrays and nanotip formation. This technique uses pinpoint, multiple‐cell DNA injections in deep areas of brain tissues, enabling target cells to be marked by fluorescent protein (FP) expression vectors. This technique has potential for use for electrophysiological recordings and biological transfection into neuronal cells. Herein, simply pressing an NTW array delivers and expresses plasmid DNA in multiple‐cultured cells and multiple‐neuronal cells within a brain slice with reduced cell damage. Additionally, DNA transfection is demonstrated using brain cells *ex vivo* and *in vivo*. Moreover, knockdown of a critical clock gene after injecting a short hairpin RNA (shRNA) and a genome‐editing vector demonstrates the potential to genetically alter the function of living brain cells, for example, pacemaker cells of the mammalian circadian rhythms. Overall, our NTW array injection technique enables genetic and functional modification of living cells in deep brain tissue areas, both *ex vivo* and *in vivo*.

## Graphical abstract

We show that DNA can be delivered and expressed in multiple cells in brain tissues *ex vivo* and *in vivo* by simply pressing the long nanowire array to express fluorescent protein with reduced cell damage. Additionally, the nanowire‐based injection of short hairpin RNA suggests potential to genetically alter living brain cell functions, including mammalian circadian rhythms.

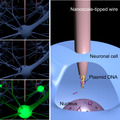

**Abbreviations**
3V: third ventricle
BF: bright field
DMEM: Dulbecco's modified Eagle's minimal essential medium
FP: fluorescent protein
JSPS: Japan Society for the Promotion of Science
JST: Japan Science and Technology
NIBB: National Institute for Basic Biology
NTW: nanoscale‐tipped wire
NW: nanowire
PI: Physik instrument
RFP: red fluorescent protein
RISC: RNA‐induced silencing complex
SCN: suprachiasmatic nucleus
SEM: scanning electron microscopy
shRNA: short hairpin RNA
TB: trypan blue
Tg: transgenic
VLS: vapor–liquid–solid

Nanoscale devices are powerful tools for monitoring and manipulating cellular physiology in organized tissue samples. Silicon nanowires (NWs) [1] and carbon nanotubes [2] have been effectively applied as intracellular nanoneedles for intracellular recordings [3] and delivery of biomolecules (e.g., DNAs and RNAs) into living cells [4, 5, 6, 7]. Such nanoneedle‐based devices enable spatial control to target a given population of cultured cells [5] and muscles *in vivo* [7].

Therefore, techniques for gene transfer into living cells have been developed to modify cell function [2, 5, 8, 9, 10, 11, 12, 13, 14, 15, 16], which can be categorized into biological, chemical, and physical methods. For example, viral infections are biological approaches, and lipofection [9] and calcium phosphate transfection [10] are chemical methods [11]. Moreover, physical methods include electroporation [12, 13], microinjections using glass pipettes [14], standard injections (SU100 Olympus) [15], atomic force microscopy [16], carbon nanotubes [2], silicon NWs [5], and gene guns [8]. Each method also has distinct characteristics, primarily based on successful gene transfer, cell survival, and functional harmless rates. Nevertheless, unlike conventional techniques, NTW array injections use transfection. Thus, (a) even though NTW array injections have transfection efficiencies comparable to viral infections and electroporation methods, they are easy to use and minimally invasive to living cells. (b) Although NTW array injections enable DNA delivery, even in neuronal cells, the transfection efficiency in neuronal cells is inferior to other cell types. (c) NTW array injections allow simultaneous DNA transfer into multiple cells due to the photolithography‐based NTW array layout.

Based on the facts above, we applied previously created NTW arrays to develop an effective gene transfer system directly into brain cells both *ex vivo* and *in vivo*, namely, a long NTW DNA stamper in this study (Figs 1 and 2A–C) [17, 18, 19]. A microwire scaffold, having a length of ~ 200 µm, served as a mechanical support in this system with sufficient stiffness for penetration. This scaffold was formed by selective vapor–liquid–solid (VLS) growth of silicon [20] (Young's modulus of 188 GPa for VLS‐grown <111> silicon microwire). Subsequently, only the tip section of the wire was sharpened through the chemical etching of silicon [17] to nanoscale dimensions (< 100 nm), thereby forming a nanotip. Moreover, in an immunohistochemical assay after injection using microneedles with a diameter 5‐µm bigger than this NTW, necrosis was marginal in rat brain cells surrounded by microglial cells [21].

**Fig. 1:**
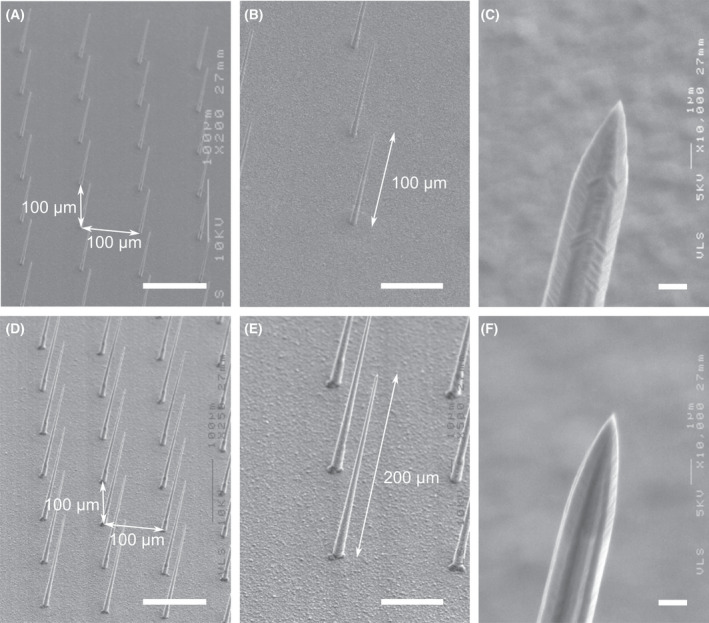
SEM images of silicon‐NTW arrays after the wire tip sharpening process. (A) 20 × 20 arrays of 100‐µm‐long silicon‐NTWs with a 100‐µm gap between the wires. Scale bar, 100 µm. (B) Individual wires in the same array are shown in (A). Scale bar, 50 µm. (C) Tip section of the same wire in (B) showing a curvature radius less than 100 nm. Scale bar, 1 µm (D–F) 20 × 20 arrays of 200‐µm‐long silicon‐NTWs. Scale bars, (D) 100 µm, (E) 50 µm, and (F) 1 µm.

**Fig. 2:**
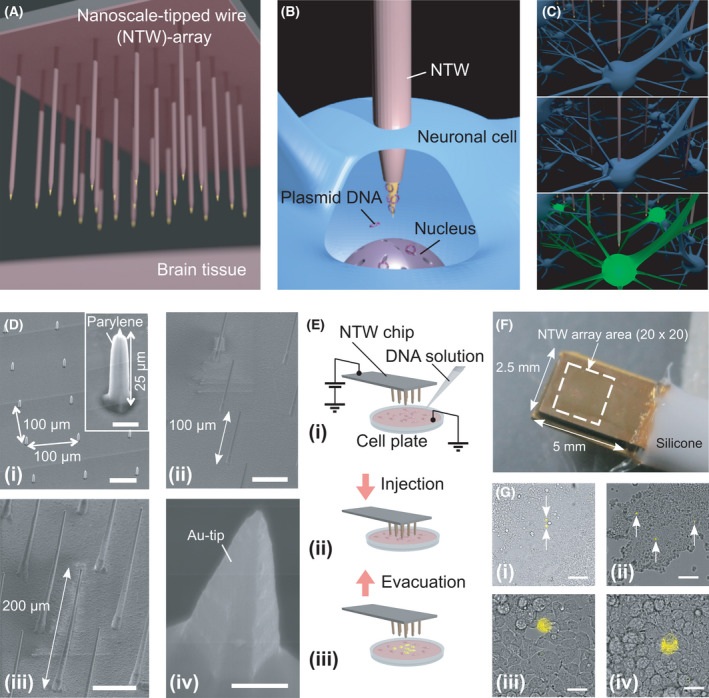
NTW arrays for delivering transgenes into multiple cells. (A–C) Schematics showing FP gene transfers using an NTW array injection. (Di) A SEM image showing an NTW array with a wire height of 25 µm. Scale bar, 50 µm. The inset shows an individual wire in the array. Scale bar, 5 µm. (Dii, Diii) SEM images of other NTW arrays with heights of (Dii) 100 µm and (Diii) 200 µm. Scale bars, 50 µm. (Div) Tip section of a wire showing the gold nanotip exposed from the parylene shell with a height of 2 µm. Scale bar, 500 nm. (E) Schematics showing gene transfers using an NTW array injection. (Ei) Image showing dropping of a solution with plasmid DNA onto cells. (Eii) Plasmid DNA injection into cells using an NTW array. (Eiii) NTW array evacuation from the cells. (F) Photograph of a packaged 20 × 20 NTW array chip electrically connected to a metal plate to provide device bias. The length of the wire is 25 µm. An NTW array chip was mounted on a manipulation system. (G) Microscope images of Venus plasmid DNA transferred into HEK293 cells using an NTW array injection. White arrows indicate HEK293 cells containing Venus plasmid DNA emitting a yellow fluorescent signal as observed through different objective lenses: (Gi, Gii) 10× and (Giii, Giv) 60×. Scale bars, (Gi, Gii) 100 µm and (Giii, Giv) 20 µm.

Herein, NTW arrays with various wire lengths were designed and fabricated to deliver DNA into cultured cells and thick brain tissue samples, *ex vivo* and *in vivo*. DNA transfections using NTW array injections were then demonstrated to modify gene expression patterns in HEK293 (human embryonic kidney) cells, fibroblast cell lines, cells in the suprachiasmatic nucleus (SCN) slice *ex vivo*, and brain tissue cells *in vivo*. For example, the molecular clock of the mammalian circadian rhythm had a 24‐h period based on clock gene expression patterns. Although the SCN of the hypothalamus is a structurally and functionally suitable brain tissue for evaluating our long NTW DNA stamper because the SCN (~ 1 mm in diameter), it contained a high density (~ 10,000 cells·mm^−3^) of neuronal cells and functioned as a mammalian central circadian clock at the apex of the regulatory hierarchy that controls the circadian rhythms [22, 23, 24] of several biochemical, physiological, and behavioral processes with approximately 24 h periods [22, 25]. Therefore, we used *Period1* (*Per1*)*:: luciferase* (*luc*) transgenic (Tg) mice to monitor rhythmic circadian changes in real time as markers of bioluminescent activity [23, 26, 27]. We also developed an *ex vivo* imaging system using SCN slices, which can be physiologically maintained in the culture for months on semidry Millicell membranes. Subsequently, slices obtained from *Per1::luc* Tg mice were used to evaluate the efficacy of gene transfer into brain cells using the long NTW DNA stamper technique. Consequently, *Per1* expression was negatively regulated by a knockdown of the transcription factor, *Bmal1* (brain and muscle Arnt‐like protein 1), after injecting the vector DNA of *Bmal1* short hairpin RNA (shRNA) into cells within the SCN slice. So, we confirmed that NTW array injections modified the biological properties of circadian rhythms via genetic manipulation.

## Methods

### Silicon microwire array synthesis

Silicon microwire arrays were synthesized through gold‐catalyzed VLS growth, using a mixed gas containing 1% PH_3_ (diluted in 99% hydrogen) and 100% Si_2_H_6_ at a gas pressure of 0.6 Pa and growth temperature of 700 °C [20].

### Plasmid DNA and shRNA vector construction

Venus (YFP having improved brightness induced by mutations) A. Miyawaki [28]. First, the CMV promoter was followed by Venus and SV40 polyadenylation sites in the pSI vector in the expression plasmid. Then, mCherry, a monomeric version of a red fluorescent protein (RFP; Clontech Laboratories, Mountain View, CA, USA), was used for each clear emission signal. Additionally, Cosmo Bio Co., Ltd. (Tokyo, Japan) provided scRNA as another control.

Afterward, shRNA was cut into siRNAs (small interfering RNAs) by the enzyme Dicer, which binds to the RNA‐induced silencing complex (RISC). RISC then attaches to the complementary sequence in the target mRNA, causing mRNA cleavage and functional knockdown. We also used a CMV promoter from Invitrogen (Waltham, MA, USA) to drive shRNA molecules targeted to knock down a specific gene in the shRNA vector.

*Bmal1* is a transcription factor and an indispensable clock factor that regulates mammalian circadian rhythms. Transcription of another clock gene, *Per1*, was induced by a heterodimer of the BMAL1 and CLOCK proteins because *Bmal1* knockout mice exhibited an arrhythmic phenotype (i.e., no circadian rhythms) [29, 30].

Here, the shRNA targeting the clock gene, *Bmal1*, was designed and cloned into a shRNA vector (Invitrogen) containing a GFP marker signal. The 21‐mers were also designed against the *Bmal1* sense strand: CCUCAUGGAAGGUUA GAAUAU Similarly, using *Clock* shRNA against the *Clock* sense strand: GAACAUCAGGCUAUGAUUACU, which is another transcription factor, shRNA significantly suppressed the expression of the critical clock gene, *Per1* [31]. Nevertheless, when NTWs were injected once into the brain's surface *in vivo*, although some could not approach due to the curved surface of the brain, they remained unbroken or bent. Besides, it is crucial to note that most NTWs were inserted into the brain *in vivo* using a manipulator, which effectively injects the brain *in vivo*. Furthermore, the brain's surface was soft enough for injection with NTWs because the dura mater on the brain's surface was removed. Subsequently, two genome‐editing vectors were purchased from Addgene (Watertown, MA, USA) after the kind permission of M. Igawa [pX330‐Cetn1/1 (Addgene #50718), pCAG‐EGxxFP‐Cetn1 (Addgene#50717)]. However, pX330‐Cetn1/1 (Addgene plasmid #50718; http://n2t.net/addgene:50718; RRID:Addgene_50718) [32] and pCAG‐EGxxFP‐Cetn1 (Addgene plasmid #50717; http://n2t.net/addgene:50717; RRID:Addgene_50717) [32] were gifts from M. Ikawa.

### Cell preparation, transfection, and injected transgene by NTW array injections

HEK293 cell line and JCRB cell line (human fibroblast) were cultured on glass coverslips (Matsunami Glass Ind., Ltd., Kishiwada, Japan) in Dulbecco's modified Eagle's minimal essential medium (DMEM; HEK293, high glucose; JCRB, low glucose) containing 10% FBS (Thermo Scientific, Waltham, MA, USA) and antibiotics (25 units mL^−1^ penicillin, 25 μg mL^−1^ streptomycin; GIBCO, Dun Laoghaire, Co Dublin, Ireland). These coverslips with cells were then transferred to PBS and placed on a glass plate by pressing with an NTW array, and covered with Venus plasmid DNA encoding FP Venus or *Bmal1* shRNA::GFP plasmid DNA or a genome‐editing vector pair in a PBS solution. Subsequently, these cells were injected with transgene DNA after 24–48 h using the NTW array. When 250 ng·µL^−1^ DNA plasmid was dropped on the cells after removing the culture medium, DNA with a negative charge was efficiently attached to the NTW tips with a positive bias of 1 V (direct current). However, when injecting with the manipulator, the more DNA with negative charge was concentrated on the top of NTWs by the positive charge, the more DNA entered cells without electrical charge. Therefore, DNA was injected into cells using NTWs using a manipulator for 1 s with no charge to detach DNA from NTWs. Additionally, 30 µL DNA solution was scattered over the SCN slice without a charge, after which 200–250 ng·µL^−1^ of DNA plasmid was injected into deep cells within the SCN slice by inserting 400 NTWs for 2 s into the SCN slice four times.

It is, therefore, possible that the DNA molecules attached to the NTW tip were partly injected into cells through NTWs, causing them to enter a cell physically or partly by endocytosis. However, this study did not distinguish between these pathways, i.e., physical transit through the cell membrane (injection) or endocytosis.

For uniform penetration conditions during gene transfers, NTW array injections containing either cultured cells or brain slices were placed on a glass plate, after which they were vertically moved with a constant displacement of 100 µm using a piezo actuator stage (PI P611ZS, Physik Instrument (PI) GmbH & Co. KG, Auf der Roemerstrasse, Karlsruhe, Germany).

After the NTW array injection, cells were observed using a fluorescent microscope (IX81, Olympus, Shinjuku, Japan) equipped with an objective lens (UFlanSApo 10× and PlanApoN 60×, Olympus), a blue LED for excitation (BMC, Tokyo, Japan), and a CCD camera (ImagEM, Hamamatsu Photonics K.K., Hamamatsu, Shizuoka, Japa), followed by imaging analysis using metamorph software (Molecular Devices, San Jose, CA, USA).

Subsequently, NIH3T3 cells of the fibroblast cell line were incubated in DMEM (low glucose) with 10% FBS (Thermo Scientific) and antibiotics (25 units mL^−1^ penicillin, 25 μg·mL^−1^ streptomycin; GIBCO) in 24‐well plates. Afterward, each plasmid DNA (0.5 mg·L^−1^) of *Per1::luc*‐expressioning vector (Promega Corporation, Madison, WI, USA), *Bmal1*, or the *Clock* shRNA expression vector (Invitrogen) was transfected into NIH3T3 cells using a lipofectamine 3000 reagent (Invitrogen; *n* = 4). After 2 days of incubation, luciferases' bioluminescence was measured using a microplate reader (Tecan, Seestrasse, Männedorf, Switzerland) in a medium containing 50 µm final concentration. Beetle Luciferin, Potassium Salt (Promega).

### Animal and SCN brain slice preparation

*Per1* *::* *luc* Tg mice of the C57BL strain, in which it was possible to monitor the circadian rhythm, were kindly provided by H. Tei (Kanazawa University) [23]. Then, we raised male and female heterozygous Tg and wild‐type mice from 6 weeks to 12 months of age under LD 12 : 12 h cycles (Light ON 8:00, Light OFF 20:00) at 22 °C. All animal studies described were kept in SPF condition and conducted in accordance with the guidelines of the Committee on Animal Care and Use of the Toyohashi University of Technology.

First, coronal sections of SCN slices (300–400‐µm thick) from wild‐type and Tg mice were cut using a micro‐slicer (Dosaka, Sakyo‐ku, Kyoto, Japan) and incubated on a culture membrane (Millicell‐CM; Millipore, Burlington, MA, USA) at a diameter of 35 mm in a petri dish containing 1.2 mL culture medium (serum‐free DMEM; Invitrogen). The culture medium was supplemented with 10 mm HEPES (pH 7.2; Invitrogen), B27 (2%; Life Technologies, Carlsbad, CA, USA), 0.1 mm luciferin (Promega), and antibiotics (25 U·mL^−1^ penicillin and 25 mg·mL^−1^ streptomycin; Invitrogen). After, the overall health of the cells in both culture dishes and SCN slices was monitored by staining with Trypan Blue (TB; in this assay, dead cells were stained blue).

### Observing transgene expression in slices

Plasmids encoding approximately 200 ng·µL^−1^ FP (Venus) and shRNA vectors (50 µL) were scattered on 400‐µm‐thick SCN slices and injected into the slices using an NTW array applied on the slices four times using a manipulator while staggering the position by 100 µm. Subsequently, we injected GFP plasmid DNA onto the slice and could estimate how deep the tip of NTW approached the injected cells, using GFP signals based on the scattering of orange fluorescent beads that marked the surface of the tissue. Illumination was provided by lasers at 488 nm (Argon) and 543 nm (He/Ne; LSM700, Carl Zeiss, Oberkochen, Germany). Data analysis was then conducted using imaris software (Zeiss). This NTW was in the micrometer range at its edge and base, preventing invasiveness while injecting deeply within samples because NTWs at high density were designed to approach numerous cells. Therefore, the efficiency of NTW injection was calculated by visually counting the number of cells with a fluorescent signal, corresponding to 3.6% (58/1600) when new 400 NTWs were initially injected into the SCN slice four times. We acknowledge that damage to the NTW after insertion into samples and the number of sharp NTWs before insertion can affect transfection efficiency. However, the specificities of each NTW were not validated by scanning electron microscopy (SEM) imaging before and after insertion into the SCN slice. Nevertheless, plasmid DNA could drop from the brain tissue surface if not inserted deeply into the brain with NTW. Bioluminescence from the SCN slices was detected by Kronos (Atto, Tokyo, Japan) while incubating at 36 °C and integrating over 20‐s intervals using a photomultiplier tube photon counting.

### *In vivo* injection of the FP into the whole brain

Fluorescent protein was also injected into neuronal cells of whole brains *in vivo* using a 200‐µm‐long NTW‐like array on SCN slices *in vitro*, after which mice (male, 20–30 g) were gas anesthetized with isoflurane (*n* = 3) to prepare them for the *in vivo* brain injection. First, the mouse's head was fixed with a stereotaxic apparatus while the gas anesthesia was administered (SR‐50; Narishige, Tokyo, Japan). Next, the cranium and dura mater of the injected potions were removed. The FP solution (Venus, concentration: 500 ng·mL^−1^) in the PBS buffer or scRNA (Cosmo Bio. Co., 200 pmol) was then dropped on the brain's surface and NTW array before injection. The NTW array was then positioned over the brain using a micromanipulator (MO‐10; Narishige), and the injection into the brain tissue was conducted four times. When NTW was inserted perpendicularly to the surface of the curved brain, less NTW was buckled and damaged after insertion into the brain tissue. After injection, sponge sheets (Spongel; Astellas Pharma, Tokyo, Japan) were placed on the injected areas of the brain to protect against damage from the external environment. Then, the scalp was closed using a surgical thread. After 2 days, the brain was removed from the mouse and fixed using formalin (4% formaldehyde). Then, the formalin‐fixed brain was cleared using SeeDB [33] for 6–8 d. Subsequently, we used two‐photon excitation microscopy (950 nm, excitation wavelength: 900 nm) for fluorescent observations at 5‐µm intervals in the z‐direction pitch, then we conducted data analysis using imaris software.

### Cosinor analyses of *Per1::luc* emission rhythms in SCN slices

We used software to assess emission rhythms of SCN slices in *Per1::luc* Tg, employing the cosinor‐fitting approach [8, 16] (Kai‐Seki Ninja SL00‐01; Churitsu Electric Corporation, Nagoya, Aichi, Japan; *n* = 3–4). The amplitude and period length of emission rhythms were then calculated as the average ratios of the peak to the trough and the average duration of single rhythmic cycle in a fitting curve. Finally, the error between the fitting curve and raw data was estimated.

### Immunohistochemistry

Mice were anesthetized and perfused with 4% paraformaldehyde in a 0.1 m PBS buffer at ZT8 (4 PM). The whole brain was kept in 4% paraformaldehyde at 4 °C for several days, after which some slice sections with 150 µm thickness that contained the SCN were cut in the coronal section using a micro‐slicer (DTK‐1000N; Dosaka).

Subsequently, immunofluorescence microscopy was conducted to detect *Per1* expression using GFP reporter signals (in *Per1* *::*GFP Tg brain slice), after which intact neurons were immunostained using a 1 : 500 dilution of anti‐rat NeuN (ab279297; Abcam, Biomedical Campus, Cambridge, UK) primary antibody, followed by a donkey anti‐rat IgG (Alexa Fluor^®^ 647; ab279297; Abcam) secondary antibody at a 1 : 500 dilution. Afterward, nuclei were stained with DAPI (DOTTIE LD034, DOJINDO, Kumamoto, Japan) at a 1 : 25,000 dilution, followed by observation of bright field (BF), GFP, Alexa Fluor 647, and DAPI images using confocal microscopy (A1; Nikon, Tokyo, Japan) with 10× or 20× magnification lenses.

### Statistics

Significant differences were indicated as *P < 0.05 by a Dunnett's *post hoc* test compared with the control using free software r software (The R Foundation, Toukei Kagaku Kenkyujo, Co., Ltd., Tokyo, Japan). Dunnett’s test compared luciferases' rhythmicity differences between the control and shRNA‐injected groups. Statistical significance was set at ***P < 0.001, **P < 0.01, *P < 0.05. However, buffer and scRNA controls were not assessed for statistical significance in period or error from cosinor fitting of *Per1::luc* emission rhythms using an unpaired Student's *t*‐test or Welch two Sample *t*‐test. Statistical significance was set at *P* < 0.05. All data are expressed as mean ± SD (*n* = 3–6).

## Results

### Fabrication of nanoscale‐tipped microwire arrays

We used vertically aligned three‐dimensional NTW arrays with a high aspect ratio for transgene injections in multiple cells. Therefore, NTW arrays were fabricated through the gold‐catalyzed VLS growth of silicon microwire arrays [20], followed by nanotip formation through batch‐processed chemical etching of silicon [17] (Fig. 1). Although typical process parameters used to form 100‐µm‐long silicon‐NTW arrays produced wire lengths ranging from 98.5 µm to 100.9 µm, conditions for 200‐µm‐long wire arrays yielded lengths ranging from 205.0 µm to 210.5 µm. Individual wires were spaced 100 µm apart. The photolithographic patterning of the catalytic gold dots also defined the sizes [17, 20]. These wires were then metalized with a 100‐nm‐thick gold by sputtering at the multisite DNA trap using an NTW array, after which they were encapsulated with an insulating layer of parylene‐C (1 µm), excluding the tip sections [18, 19].

A highly accurate VLS growth rate of 1 µm·min^−1^ produced wires with varying lengths [20]. These wires were applied to different biological samples for DNA transfer. Thus, each NTW in the array could reach individual cell bodies in the neuronal circuits. Therefore, we designed three distinct lengths of wire arrays; 25‐µm‐long wires (Fig. 2Di) for cultured cells, 100‐µm‐long wires (Fig. 2Dii), and 200‐µm‐long wires (Fig. 2Diii) for thick tissue samples, including *ex vivo* brain slices (SCN) and *in vivo* brain tissue samples. Fig. 2Div shows a SEM image of a typical wire tip exposed from the ~ 1‐µm‐thick parylene shell. The fabricated NTW had a tip diameter of < 100 nm. Similarly, transmission electron microscope observations confirmed that the curvature radius of the tip was 150 nm [17]. In subsequent studies, we biologically evaluated these three distinct wire array lengths as a device for gene transfer.

### Injection of a reporter plasmid DNA encoding a fluorescent protein, Venus, into HEK293 cells

We first tested a 25‐µm‐long wire array using cultured cells. Then, a plasmid DNA carrying a fluorescent gene‐encoding protein, Venus (an YFP variant), was injected into HEK293 cell lines using an NTW array (wire length, 25 µm; the gap between the wires, 100 µm; the number of wires on the device was 400 in a 20 × 20 array) with a positive device bias of 1 V (Fig. 2E). Next, the fabricated NTW array chip mounted on a manipulation system was used for cell injections (Fig. 2F). Then, cells expressing Venus yellow fluorescent signals were observed a day after injection (Fig. 2G).

The efficiency of cell death associated with the NTW array injection was quantitatively determined from the number of fibroblast cells stained using TB (Fig. S1). As observed, the NTW array injection caused so little cell death (imaris software), showing that it was not toxic to living cells. NTW‐injected cells also displayed a normal morphology when examined using Venus fluorescent signal, with little TB (a marker of dead cells) co‐localization. This is because that the piezo actuator (see Methods) enabled accurate injections at high speed (376 µm·s^−1^). The other reason is that the tip diameter of the NTW was thin enough (100 nm) not to rupture cell membranes. Furthermore, the base of this NTW was not particularly effective in brain tissue, considering the low density of neuronal soma at the brain's surface around the base of the NTW and the damage to these cells at the surface of the brain slice induced by cutting. Therefore, a single injection of Venus plasmid DNA using the 25‐µm‐long NTW array enabled a minimally invasive gene transfer into multiple cells. Our biological evaluation also indicated that the negative electrical bias on the NTW had a negligible relationship with NTW array injection efficiency because the adhesive force between the tissue and the NTW (gold tip) was more predominant than the attraction due to electrical charges [18]. Likewise, a previous publication confirmed that the NTW‐inserted fluorescent bead positions in agarose gel coincided with the NTW pattern on the silicon chip without damaging NTWs.

### Injection of plasmid DNA reporters into cells in brain slices *ex vivo*

**Fig. 3:**
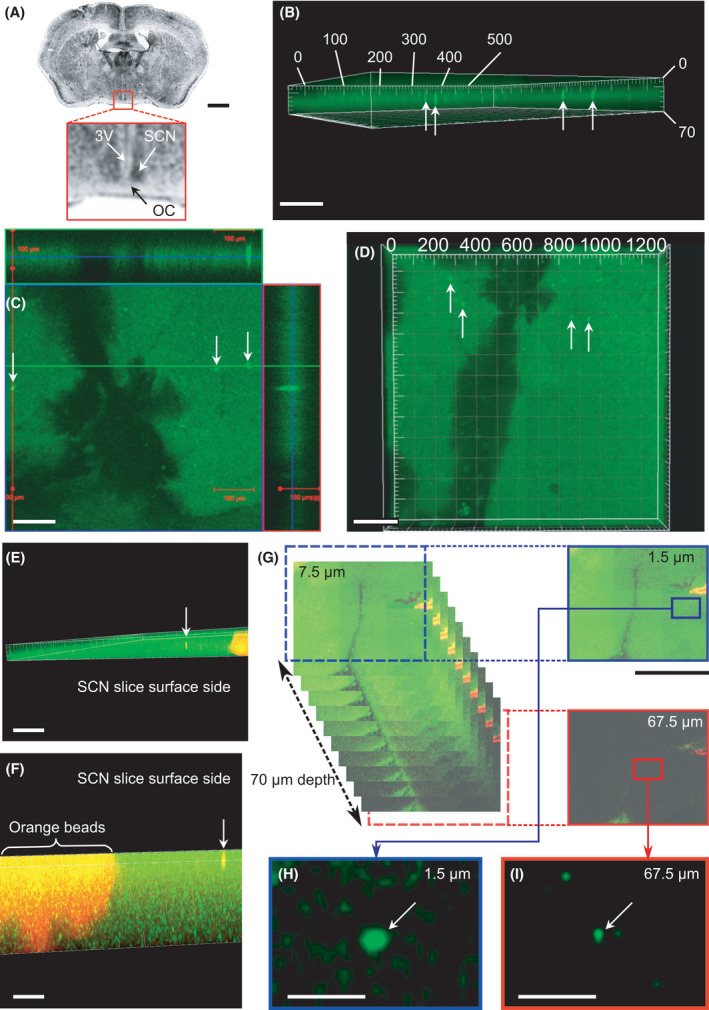
FP transgene injection into a brain slice using a 100‐µm‐height NTW array. (A) Coronal sections of the mouse brain. Scale bar, 1 mm. Red squares show the SCN, optic chiasm (OC), and third ventricle (3V). (B) Three‐dimensional imaging of the SCN slice by confocal microscopy with a 10× objective lens 2 days after the NTW array injection. White arrows indicate SCN cells containing Venus plasmid DNA molecules expressing fluorescent signals deep within the slice. Scale bar, 100 µm. (C, D) Three‐dimensional imaging of the SCN slice with Venus signals in the deep area. Scale bars, (C) 100 µm and (D) 200 µm. (E, F) Three‐dimensional images of the SCN slice with a Venus yellow signal inside the slice and an orange fluorescent signal from beads deposited onto 3V. Several cells containing Venus signals were aligned with the vertical NTW injection pathway. Scale bars, (E) 200 µm and (F) 100 µm. (G) Montage images of the SCN slice subjected to NTW injections along the z‐axis of each 3 µm Z stack. Scale bar, 1 mm. (H, I) Enlarged images of injected cells at 1.5 µm and 67.5 µm depths taken from the montage images of (G). The FP transgene was injected deep into the cells (~ 70 µm below the surface of the slice). Scale bars, (H, I) 100 µm.

Next, we evaluated a 100‐µm‐long wire array using brain slices *ex vivo*. The Venus‐expressing plasmid DNA was concentrated on the tip of the 100‐µm‐long NTW by applying the same positive bias of 1 V. The FP transgene was subsequently injected into the cells within the SCN slice (see Methods, Fig. 3A) using the NTW array. The 300–400‐µm‐thick slices were incubated at 36 °C for approximately 4 days. Then, the FP signal in several cells was observed using a confocal microscope after NTW injection with a 100‐µm long wire (Fig. 3B–D, Movie S1). We observed that cells with FP signals were located at NTW sites in a ~ 70‐µm‐deep section of the SCN slice.

Furthermore, results showed that NTW array‐based gene transfer aided pathophysiological analyses of brain tissue samples composed of multilayered neuronal cells. However, conventional NWs remained too short (< 10 µm long) to penetrate the surface tissue layer of a murine brain (a standard animal model used in brain research to reach neuronal cells successfully). Moreover, such bending or disintegration of NWs made it difficult for such NWs to penetrate tissue samples. Therefore, to access neuronal cells in a murine brain, NWs longer than several hundred microns with appropriate stiffness should be innovated.

Additionally, cells in the slice did not display fluorescent signals after NTW injection without plasmid DNA as a negative control (only buffer injection), despite setting the observation's sensitivity to reduce autofluorescent signal (Fig. S2). Therefore, we repeated the experiment using another plasmid DNA encoding RFP, mCherry, to improve the contrast of transgene‐derived fluorescent signal with autofluorescence of brain tissue samples (Fig. S3). Since the little autofluorescent signal was identified in this condition for observing mCherry, the injected cells expressing mCherry in the SCN slice were quickly confirmed. We also observed a low transfection efficiency with Venus signals in NIH3T3 cells and within the SCN slice after injection with a chip without NTWs as negative controls (the same method used with the chip having NTWs; Figs S1, S2, S4). As observed, the percentages of dead cells detected by the chip without NTWs and the injection itself were 12.16% and 16.06%, respectively, dyed using TB (Figs S1A and S5).

In slices, many cells on the surface were dyed during brain cutting, preventing an accurate estimation of living efficiency after NTW injection. However, most cells in the SCN slice were not stained with TB and remained alive 2 days after NTW injection and incubation (Fig. S2B).

We performed genome editing using a CRISPR‐Cas9 system of pX330‐Cetn1/1 and pCAG‐EGxxFP‐Cetn1 by injection on the SCN slice [32]. GFP protein was restored only when genome editing was successful in this vector system. Several cells with GFP signals were observed in the SCN slice after 2 days of incubation following DNA injections four times with a 100‐µm NTW array (Fig. S6). Therefore, targeted genome editing for functional analysis was achievable in cultured brain slices using the 100‐µm‐long wire array.

### Depth estimation of transgene delivery into cells by NTW from the brain slice surface

**Fig. 4:**
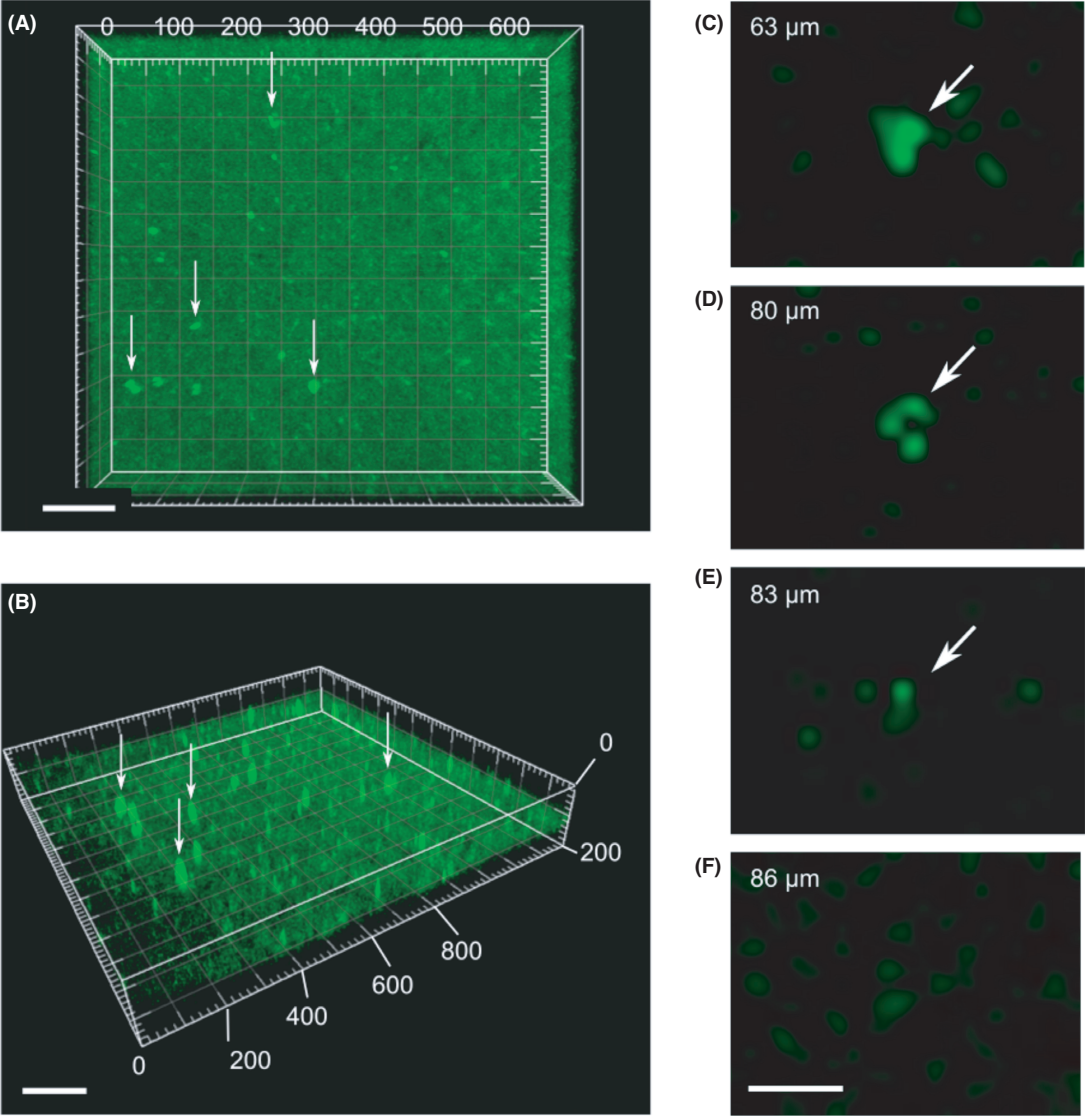
FP transgene injection into a brain slice using a 200‐µm‐long NTW array. (A, B) Three‐dimensional confocal microscopy imaging of the Venus signal in the SCN slice after injection using a 200‐µm‐long NTW array. Scale bars, 100 µm. (C–F) Enlarged images of a section of injected cells at depths ranging from 63 to 86 µm. The FP transgene can be injected more profoundly in the SCN slice using a more extended NTW array chip. The distance between the SCN slices' surface and the deepest cell of a Venus signal was more than 80 µm for a 200‐µm wire length vs ~ 70 µm for an array with a 100‐µm wire length (Fig. 3G). Scale bars, 20 µm.

To estimate how deeply located cells from the brain slice surface NTW can be associated with injected transgenes, orange fluorescent beads were dispersed on the surface of the SCN slice to measure the depth of injected cells from the slice surface before transgene delivery by NTW. Plasmids encoding approximately 200 ng·µL^−1^ FP (Venus) and shRNA vectors (50 µL) were then distributed over 400‐µm‐thick SCN slices, followed by injection into the slices using an NTW array applied on the slices four times with a manipulator while adjusting the position by 100 µm. We also injected GFP plasmid DNA onto the slice. Then, we estimated how deep the tip of NTWs approached the injected cells with GFP signals based on the scattering of orange fluorescent beads marking the tissue surface. Lasers at 488 nm (Argon) and 543 nm (He/Ne) provided illumination (LSM700; Zeiss), and data analysis was conducted using the imaris software. The most deeply located fluorescent cells reached by 100‐µm‐long NTWs were 67‐µm in depth from the surface (Fig. 3E–I, Movie S2), whereas the cells located at the deepest position attained by the 200‐µm‐long NTWs were > 83 µm in depth (Fig. 4). These findings confirmed that the NTW array injection could deliver a transgene to a deep area of an SCN brain slice.

Previously, excellent methods for delivering various molecules into cells using silicon NW arrays have been reported. These methods are highly efficient and do not require viral packaging [5]. Notably, the vertical silicon NW array density components (20,000 components at 1000 components·cm^−2^) enables high‐throughput screening. Besides, our NTW array injection, in which each wire had a long microwire scaffold (> 100 µm), can reach deep areas in the brain using three‐dimensional NTWs against NW arrays (> 10 µm) long. However, in theory, longer wires can deliver a transgene into deeper areas in the brain. For example, our NTWs (> 200 µm in length) can reach layer 2 of the cortex in mice.

### *In vivo* injection of plasmid DNA reporters into cells in the living mouse brain

**Fig. 5:**
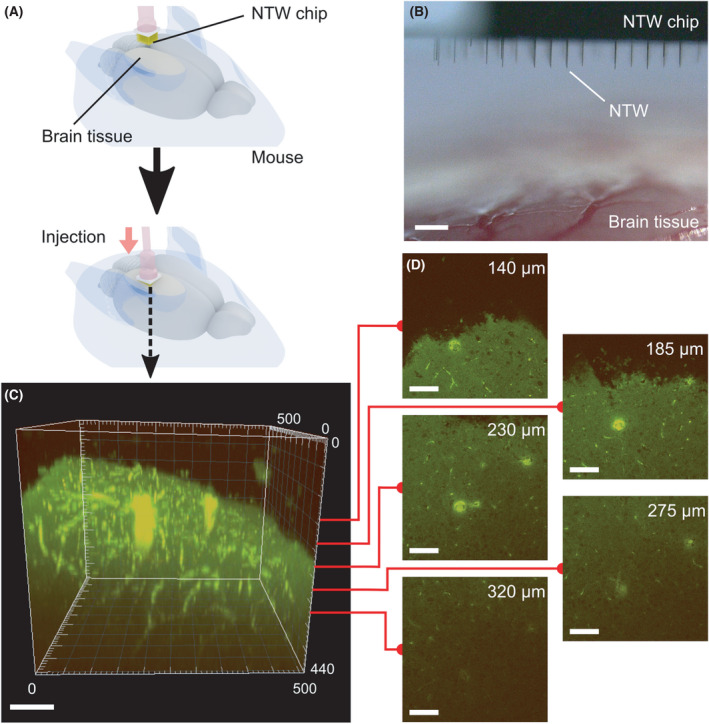
*In vivo* injection of Venus expressional vector plasmids into a brain tissue sample using a 200‐µm‐long NTW array. (A) Schematics showing the *in vivo* injection of FP into a mouse's cortex using an NTW array. The NTW array, which was held in place by a micromanipulator, penetrated the tissue's barrel area through the window of the skull. (B) A photograph showing the NTW chip positioned over the barrel area of the cortical surface. Scale bar, 200 µm. (C) Three‐dimensional images of the Venus fluorescent signal in the brain. The image was obtained from the whole brain, extracted from the mouse after the injection. Scale bar, 100 µm. (D) Z‐stack images of 140, 185, 230, 275, and 320‐µm depths obtained from the three‐dimensional images along the *z*‐axis of each 5 µm Z stack. Scale bar, 100 µm.

Furthermore, we evaluated the 200‐µm long wire array for gene transfer into neuronal cells in the brain of living mice (Fig. 5A,B). Therefore, to estimate the depth of gene transfer from the brain surface, we used whole‐brain imaging with a two‐photon laser‐scanning microscope after treating brain specimens with a tissue transparency reagent and the SeeDB method. This estimation was conducted to observe the deeper area in the brain having low background noise [34]. Hence, when the Venus‐expressing plasmid DNA was injected using an NTW length of 200 µm, we observed Venus‐positive cells even in a 200‐µm deep area of the whole brain from the surface (Fig. 5C,D, Movies S3 and S4).

### RNAi was conducted in SCN cells by the NTW array

**Fig. 6:**
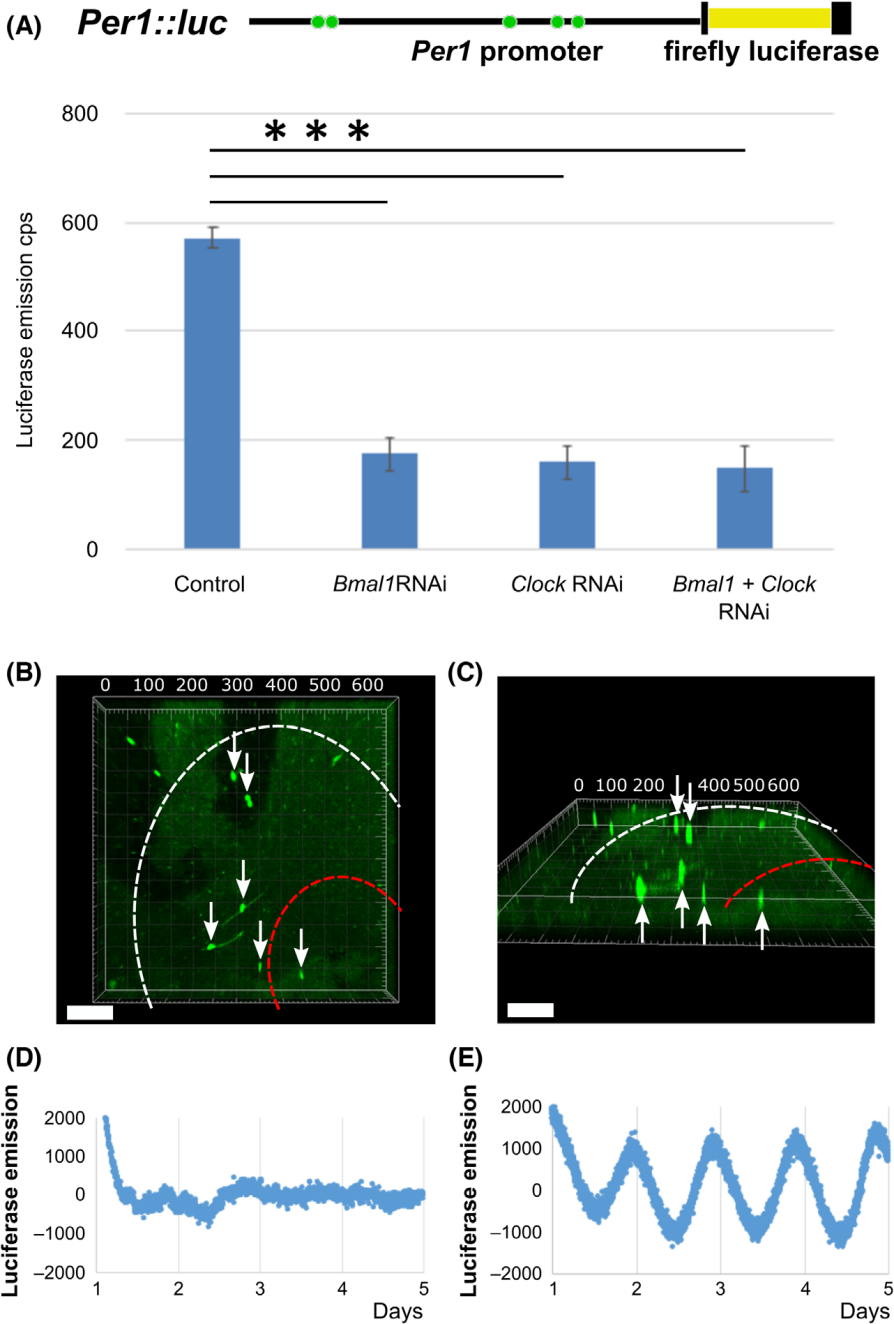
*Bmal1* shRNA vector knocks down *Per1* expression in the stable *Per1* *::* *luc* cell line and the SCN slice of *Per1* *::* *luc* Tg mouse using an NTW array. (A) The construction of *Per1::luc* Tg mice, the 6.7kb *Per1* promoter region was followed by the firefly luciferase. The five green circles indicate Ebox consensus sequences where heterodimer of BMAL1 and CLOCK transcription factors bind. Inductivity of luciferase activity driven by the *Per1* promoter was 1.2 times that induced by basal *Bmal1* or *Clock* gene expression in the *Per1* *::* *luc* cell line compared to the basal level. *Per1* *::* *luc* expression was reduced by 1/3 when the *Bmal1* or *Clock* shRNA is expressed. Each value represents the mean ± standard error of the mean (SE, *n* = 5). Control: neither shRNA is expressed; *Bmal1*RNAi: *Bmal1* shRNA is expressed; *Clock* RNAi: *Clock* shRNA is expressed; *Bmal1* + *Clock* RNAi: *Bmal1* and *Clock* shRNA are expressed. Dunnett's test was used to compare rhythmicity between knockdown groups for each RNAi and control group. Statistical significance was set to ***P < 0.0001. (B, C) Three‐dimensional images of the GFP signal for the *Bmal1* shRNA plasmid deep within the SCN slice after NTW array injections. White arrows indicate SCN cells injected with *Bmal1* shRNA using an NTW array. A white dashed circle indicates the whole area of the ventrolateral SCN, and a red dashed circle shows the core of the dorsomedial SCN. Since most *Bmal1* shRNA‐injected cells with GFP signal are identified as pacemaker neurons in the white circle area of SCN, *Per1* *::* *luc* emission oscillation of whole SCN slices from Tg mice was derived mainly from the white circle area (ventrolateral) rather than the red circle area (dorsomedial). Scale bars, 100 µm. (D, E) The luciferase emission rhythms from an injected SCN slice receiving NTW array‐based injection of *Bmal1* shRNA or only buffer, with a final concentration of 100 µm luciferin, measured during incubation of the SCN slice and detrended.

To test whether functional genetic manipulation was achievable by our NTW, we used a brain SCN slice culture system of *Per1* *::*luc Tg mice for the construction (Fig. 6A). The transgene of these mice was composed of the 6.7 kb *Per1* promoter region fused into firefly luciferase as a reporter gene, enabling us to study the circadian rhythms of living organs in real time. As transcription factors, the heterodimer of both BMAL1 and CLOCK bound onto the E box of the *Per1* promoter, allowing the *Per1* transcription to be up‐regulated [29, 30]. First, *Bmal1* or *Clock* shRNA expression vectors were transfected into *Per1::luc*‐expressing fibroblasts using lipofectamine reagent (Invitrogen, USA; *n* = 4 assays with *Per1::luc*‐expressing fibroblasts). Then, after 2 days of incubation, *Per1::luc* expression was reduced to approximately 30% of the control (transfected empty vector) upon *Bmal1* or *Clock* shRNA transfection (***P < 0.0001, Dunnett's test, SE, *n* = 5; Fig. 6A). Therefore, we used our NTW array‐based shRNA injection to knock down either of these transcription factors in SCN cells (clock pacemaker cells). Subsequently, expression vectors encoding GFP were simultaneously injected into the SCN slice of the *Per1* *::*luc Tg mice to monitor successful gene transfer to targeted cells. Knockdown of *Bmal1* in GFP‐positive SCN cells 1–3 d after injection significantly suppressed the *Per1* expression (Fig. 6B,C) [23]. These experiments indicated that multiple molecules could be delivered simultaneously into multi‐cells in a deep area of an SCN slice to manipulate the function of targeted cells, which is another advantage of NTW array injection.

### Knockdown effects of RNAi on the SCN slice using the NTW array

The gene, *Per1,* is an indispensable component of the central clock system that regulates mammalian circadian rhythms, and its transcription and translation of *Per1*oscillate autonomously in the mouse SCN [23, 24, 29, 35]. Therefore, we established a *Per1* *::* *luc* Tg mice model to monitor circadian rhythms in real time, in which bioluminescence protein firefly luciferase was rhythmically expressed under the control of the mouse *Per1* promoter. As observed, rhythmic emissions from 300–400‐µm–cultured SCN slices of *Per1* *::* *luc* Tg mice can persist for several months *in vitro* [26, 27]. An shRNA vector targeting the *Bmal1* gene or *Clock* was also confirmed to be functional [30, 34]. Both *Bmal1* and *Clock* are central clock genes that regulate circadian rhythms and act as transcription factors for the *Per1* gene [30, 34]. *Per1* expression was significantly suppressed to approximately 30%, transfected using *Bmal1* shRNA vector with a GFP marker in *Per1* *::* *luc* cells *in vitro* (Fig. 6A). The *Bmal1* shRNA vector was introduced into an SCN slice from a *Per1* *::* *luc* Tg mouse using an NTW array injection. After injecting the *Bmal1* shRNA vector into numerous cells of the SCN slice in *Per1* *::* *luc* Tg mouse using an NTW array injection, the 24‐h bioluminescence *Per1* emission rhythms were disturbed, compared to those observed after injecting only the PBS buffer by the NTW array injection: Also, we observed that *Bmal1* shRNA knockdown had *Per1* expressional rhythms disturbed in the SCN with a more extended period (Fig. 6D,E; *n* = 3). Therefore, providing a possible explanation, *Per1* *::* *luc* rhythms in the SCN slice displayed diminished amplitude of emission oscillation in some pacemaker cells of the SCN that showed GFP signal when injecting *Bmal1* shRNA. Similarly, a shRNA vector targeting the *Clock* gene bound BMAL1 as a heterodimer for the *Per1* gene transcription factor (Fig. S7A–C; *n* = 3). The bioluminescent rhythms were cosinor fitted using the least‐squares method (Ninja). Moreover, the SCN slice injected with *Bmal1* or *Clock* shRNA had a more extended period (τ) beyond 24 h, with the error of fitting a cosinor model being compared to only the buffer (Fig. S7D,E).

Although both *Bmal1* and *Clock* genes were transcription factors of *Per1, Bmal1* was considered a more critical driving force in determining circadian rhythms based on a previous study using knockout mice. In this study, the knockdown of the *Bmal1* gene was shown to severely repress *Per1* expression rhythms in Fig. S7B compared with clock genes in Fig. S7C upon injection of shRNA vector by NTW. Moreover, even though *Bmal1* shRNA injection with the NTW array did not cause complete arrhythmia (lacking steady rhythms) in the SCN slice, effects on *Per1* *::* *luc* emission rhythms are proposed to depend on the identity and percentage of pacemaker cells injected with *Bmal1* shRNA.

## Discussion

The NTW device application to genetic modification of living neuronal cells within brain tissue samples *ex vivo* and *in vivo* plays a critical role in neuroscience. In this study, we report that an array of NTWs with a length of 200 μm, which was 40‐fold longer than conventional ones, was facilitated for *ex vivo* and *in vivo* applications to cells in thick brain tissue samples. Therefore, the fabricated three‐dimensional NTW array provided a sufficient DNA “stamper” to deliver transgenes into multiple cells deep within brain tissue samples, *ex vivo* and *in vivo*. NTW arrays can simultaneously deliver transgenes into multiple specific SCN cells at the surface and deep area of the slice, corresponding to the NTW layout (e.g., 20 × 20 array). Such NTW arrays can then be used to conduct intracellular electrophysiological recordings, contributing to the study of neuronal networks [19, 34, 36]. Therefore, to determine the neuronal cell to measure, recorded neuronal cells by NTW were marked and identified with fluorescent signals using the NTW injection of the FP transgene. This method helped determine which neuronal circuits corresponded to specific physiological functions in deep brain areas.

For cells that penetrated with NTWs, it is assumed that some cells die when their cell membrane is pierced. However, specific cells with fluorescent signals survived after the tip of the NTW was temporarily inserted. Besides, it is difficult to identify blunt (not sharp) or bent NTWs before or after injecting them into brain tissue samples. Therefore, while adjusting the position by 100 µm using a manipulator (Fig. S8A), we injected a 250 ng·µL^−1^ RFP code plasmid DNA into an SCN slice on a 1% agarose gel PBS buffer at six points using a single NTW of 200 µm. These positions with red fluorescent signals coincided with those injected by the NTW tip. Furthermore, six‐cell positions with red fluorescent signals (white arrows) in the SCN slice were also detected in one line at approximately 100 µm intervals within a wide field of view after incubating the SCN slice for 2 days (Fig. S8B). Hence, since these 6‐cell positions with GFP fluorescent signals of *Per1* *::*GFP Tg slice were observed with a yellow signal in the merged image of Fig. S8C, injected cells at the six points were identified as neuronal cells (Fig. S8B,C).

NTW injection techniques can be applied to deliver transgenes into deep tissue areas *in vivo*. The cells injected by NTW with Venus signal can be found at the deepest position of approximately 80 µm with 100‐µm‐long NTWs. However, injected cells using NTW with the Venus signal and 200‐µm‐long NTWs can be located at a deeper location (roughly 150 µm) than the 100‐µm‐long NTW. Therefore, NTW's ability to be extended over 400‐µm in length with longer silicon crystal growth [36], enabling transgenes to be injected in deeper positions of the brain both *in vitro* and *in vivo*.

Although the transgene is delivered into cells physically by NTWs like a cantilever in an injection system (Su100; Olympus), endocytosis can contribute to gene transfer by NTW's stimulation to cells [37]. NTW‐array injection techniques provide novel means to alter the function of living cells in the brain genetically. We expect that this technique applies to electrophysiological and genetic studies and can support patients with neurological diseases by restoring and compensating the function of an injured neuronal circuit. Moreover, intracellular electrophysiological recordings have been crucial in the study of neuronal networks [3]. Therefore, since we have demonstrated the intracellular recording capability of our NTW [19, 36], our injection technique allows identifying electrically recorded cells marked by fluorescent signals and modifying their gene function with RNAi knockdown (injection of shRNA), favoring genome editing (injection of CRISPR elements). Hence, this methodology will help to understand neuronal circuits further, clarifying the connectivity map of the brain.

At this sampling time (daytime ZT8, 8 h after light on), *Per1's* gene expression was so high in neuronal cells of the SCN in *Per1* *::*GFP Tg mice that GFP signals were strongly detected in neuronal cells of the SCN. In immunostaining experiment, the nuclei of neuronal cells in the SCN slice were specifically stained using red fluorescence signals (Alexa Fluor647) with an anti‐NeuN primary antibody first (anti‐rat NeuN antibody; Abcam), followed by an Alexa Fluor 647 (donkey anti‐rat IgG; Abcam) secondary antibody. In contrast, the nuclei of all cells were stained using DAPI (DOJINDO). BF, GFP, Alexa Fluor647, and DAPI images were then observed using confocal microscopy (A1, Nikon) with a 10× or 20× magnification lens (Fig. S9). In the SCN slice of *Per1* *::*GFP Tg mice, many yellow cells were co‐stained with Alexa Fluor 647 and GFP signals within the SCN (Fig. S9). Therefore, most NTWs approached these yellow cells as neuronal cells in merged images. It has been previously reported that SCN comprises 20,000 neuronal cells, including some glial cells, such as astrocytes. Thus, a high probability exists that several NTWs would approach neuronal cells among the 1600 points (maximally) within the SCN when 400 NTWs were injected into the SCN slice four times.

Two different characteristic parts constitute the SCN: the pacemaker cells are located in the shell area, and the input cells are located in the core area by neuronal projection. Several cells with disturbed rhythms caused by *Bmal1* shRNA injection can therefore be inclined in the shell area of the SCN containing the pacemaker cells. Hence, *Per1* *::* *luc* emission rhythms of whole SCN sliced sections indicated more diminished circadian rhythms in some pacemaker cells knocked down by injecting *Bmal1* shRNA. In contrast, the *Per1* *::* *luc* emission rhythms of the SCN slice, using the *Bmal1* shRNA injection, did not inhibit circadian rhythms. This failure was partly because *Bmal1* knockdown cells present insufficiently perturbed the pacemaker function in SCN. Quantitively, NTW approached too limited cells (400 × 4 = 1600 cells, neuronal cells or not) among the approximately 10,000 neuronal cells in the SCN slice to disrupt circadian rhythms. This disruption was partly because groups of neuronal cells without *Bmal1* shRNA orchestrated circadian rhythms within the whole SCN. Additionally, the SCN slice maintained luciferase emission rhythms after administering the NTW injection (not NTW array injection) four times and after adding only the buffer on the slice (Fig. 6E). These observations excluded the possibility that the long brain slice culture or physical tissue damage by NTW affected analyses of the luciferase emission rhythms. It was also confirmed that the SCN slice was well cultured because the culture medium exchange recovered the luciferase emission rhythms. Consequently, our experimental findings demonstrated that although injecting cells with *Bmal1* shRNA did not result in critical arrhythmia, it diminished the circadian rhythm system. Moreover, the bioluminescent rhythm in the SCN slice of *Per1::luc* Tg injected with *Bmal1* or *Clock* shRNA indicated diminished circadian rhythm estimated by cosinor fitting with a more extended period of higher error compared to the control injected with only PBS buffer (Fig. S7). Additionally, an established rhythmic emission of SCN slice in *Per1::luc* Tg was observed after injection by NTW with scRNA (random sequence), similar to the PBS buffer results. However, the period and error of rhythmic emission in scRNA and PBS conditions were insignificantly different (*P* > 0.05 after the *t*‐test; Fig. S10).

It has been observed that the *Bmal1* gene significantly determined circadian rhythms since *Bmal1* knockout mice exhibited a severe phenotype with arrhythmia (no rhythmic activity), inhibiting circadian rhythms. Likewise, in this study, *Bmal1* knockdown repressed the SCN display of circadian rhythms upon injecting the RNAi plasmid into the pacemaker cell in the SCN. This repression was because the RNAi plasmid used for knockdown was effectively injected into not all cells, but parts of cells among the SCN shell region, which contains most pacemaker neurons (between white dotted line and red dotted line; Fig. 6B,C).

Silicon growth technique–based three‐dimensional and high aspect ratio NTW arrays provide adequate and low invasive DNA stampers for delivering transgenes into multiple cells among the deep area of brain tissue samples *ex vivo* and *in vivo*. This NTW technique effectively performs neuronal circuit investigations with *in vivo*, *ex vivo*, and *in vitro* injections. For example, this technique can be used for both loss of function (RNAi, genome editing) and gain of function (overexpression of transgene) experiments. Therefore, our NTW array injection technique has opened a new field where DNA injections can alter the genetic function of living cells within deep areas of brain tissue samples by labeling cells with FPs. This technique also favors functional knockdown by RNAi, including knockout and knock‐in experiments via genome editing. Furthermore, it applies to several electrophysiological and genetic studies, using the intracellular electrophysiological recording to mark approaching cells and perform transgene injection, followed by a long‐term electrophysiological recording. Hence, applying our NTW‐based injection to brain tissue samples can provide a novel and effective method for analyzing the “population effects” involved in brain function maintenance. Additionally, it can support patients with neurological diseases by restoring and compensating the function of injured neuronal circuits.

## Conflict of interest

The authors declare no conflict of interest.

## Author contributions

RN and TK conducted each experiment, analyzed data, performed statistical analyses, prepared figures, and contributed to manuscript writing. MM assisted in experiments, whereas AG, YK, HS, and SY manufactured, conducted experiments, and analyzed. We are also grateful to TI, HT, and MI for their supervision and helpful discussion.

## Supporting information


**Fig. S1.** Venus transgenes injected into fibroblast cells by a chip with and without NTWs.
**Fig. S2.** NTW array injection is not toxic to the SCN slice.
**Fig. S3.** Red FP (mCherry) transgene injected into the SCN slice.
**Fig. S4.** Venus transgenes injected into the SCN slice of wild‐type mice using chips without NTWs.
**Fig. S5.** Fibroblast cells dyed using TB after injection by chip without NTWs, or without injection itself.
**Fig. S6.** Genome editing of cells in the SCN slice by CRISPR‐Cas9 system with an NTW array.
**Fig. S7.** Luciferase emission rhythms of the SCN slice after NTW array RNAi injections.
**Fig. S8.** RFP plasmid DNA molecules injected into the SCN sliced sections of *Per1* *::*GFP Tg mice by a single NTW.
**Fig. S9.** Immunohistochemical images of the SCN slice in *Per1* *::*GFP Tg mice at ZT8.
**Fig. S10.** Luciferase emission rhythms of the SCN slice after NTW array scRNA injections.

**Movie S1.** Fluorescent imaging of an SCN slice injected with Venus plasmid DNA using an NTW array.

**Movie S2.** Fluorescent imaging of an SCN slice injected with Venus plasmid DNA using an NTW array and subsequent labeling with orange fluorescent beads.

**Movie S3.** Observation of the barrel area deep in the whole mouse brain using a two‐photon laser‐scanning microscope after an *in vivo* injection with Venus plasmid DNA using an NTW array.

**Movie S4.** A one‐time administration of NTW injections into the brain's surface *in vivo*.

## Data Availability

The data that support the findings of this study are available from the corresponding author upon reasonable request.
