# Supplementary material for: Nanoscale‐tipped wire array injections transfer DNA directly into brain cells ex vivo and in vivo
Source: FEBS Open Bio. 2022 Mar 15;12(4):835–51. doi: 10.1002/2211-5463.13377 (PMC8972050; doi:10.1002/2211-5463.13377)
Supplement: Supplementary file 1 — Fig. S1. Venus transgenes injected into fibroblast cells by a chip with and without NTWs. Fig. S2. NTW array injection is not toxic to the SCN slice. Fig. S3. Red FP (mCherry) transgene injected into the SCN slice. Fig. S4. Venus transgenes injected into the SCN slice of wild‐type mice using chips without NTWs. Fig. S5. Fibroblast cells dyed using TB after injection by chip without NTWs, or without injection itself. Fig. S6. Genome editing of cells in the SCN slice by CRISPR‐Cas9 system with an NTW array. Fig. S7. Luciferase emission rhythms of the SCN slice after NTW array RNAi injections. Fig. S8. RFP plasmid DNA molecules injected into the SCN sliced sections of Per1 ::GFP Tg mice by a single NTW. Fig. S9. Immunohistochemical images of the SCN slice in Per1 ::GFP Tg mice at ZT8. Fig. S10. Luciferase emission rhythms of the SCN slice after NTW array scRNA injections. [file FEB4-12-835-s003.docx]

**Supplementary information**

**Supplementary information titles and legends**

Figure S1. Venus transgenes injected into fibroblast cells by a chip with and without NTWs.

Figure S2. NTW-array injection is not toxic to the SCN slice.

Figure S3. Red FP (mCherry) transgene injected into the SCN slice.

Figure S4. Venus transgenes injected into the SCN slice of wild-type mice using chips without NTWs.

Figure S5. Fibroblast cells dyed using TB after injection by chip without NTWs, or without injection itself.

Figure S6. Genome editing of cells in the SCN slice by CRISPR-Cas9 system with an NTW array.

Figure S7. Luciferase emission rhythms of the SCN slice after NTW-array RNAi injections.

Figure S8. RFP plasmid DNA molecules injected into the SCN sliced sections of *Per1::*GFP Tg mice by a single NTW

Figure S9. Immunohistochemical images of the SCN slice in *Per1::*GFP Tg mice at ZT8

Figure S10. Luciferase emission rhythms of the SCN slice after NTW-array scRNA injections.

Movie S1. Fluorescent imaging of an SCN slice injected with Venus plasmid DNA using an NTW array.

Movie S2. Fluorescent imaging of an SCN slice injected with Venus plasmid DNA using an NTW array and subsequent labeling with orange fluorescent beads.

Movie S3. Observation of the barrel area deep in the whole mouse brain using a two-photon laser-scanning microscope after an *in vivo* injection with Venus plasmid DNA using an NTW array.

Movie S4. A one-time administration of NTW injections into the brain's surface *in vivo.*

**Figure S1. Venus transgenes injected into fibroblast cells** **using a chip with and without NTWs.**

(A) Fibroblast cells with a fluorescent signal injected Venus plasmid DNA using a chip with NTWs that were estimated against four types of negative controls. BF image and Venus of fibroblast cells injected Venus plasmid DNA or PBS buffer by chip without NTW (no bias condition) or no injection. Scale bar: 20 µm. (B) The fluorescent signal in all negative controls was lower than that in the positive control (SD, n = 10). Significant differences are depicted as *p* < 10^−7^ *** by Dunnett's post hoc test among negative control samples to positive samples of the injected Venus DNA by a chip with NTWs (R software). Nega1: DNA injected using chip without NTWs, Nega2: DNA not injected using a chip, Nega3: no DNA injected by chip without NTWs, Nega4: no DNA not injected using a chip.


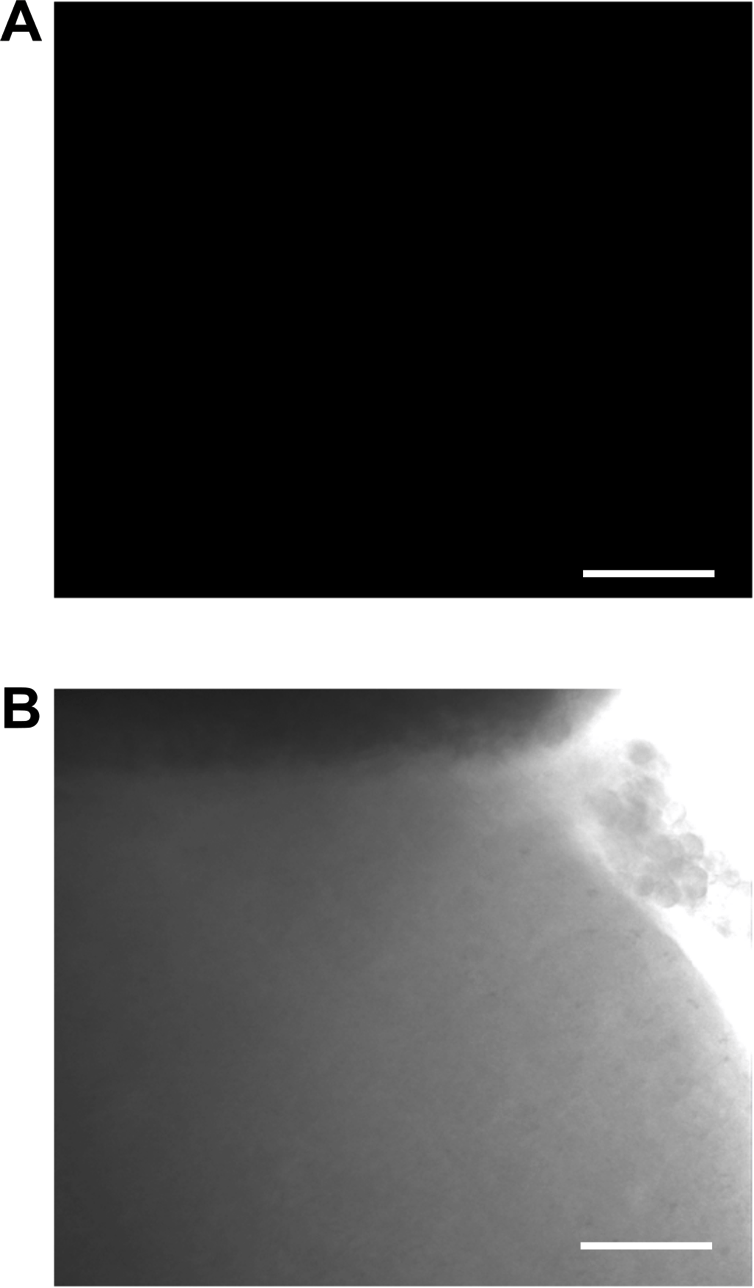


**Figure S2. NTW-array injection nontoxic to the SCN slice.**

(A) FP image of the wild-type SCN slice one day after injecting only PBS buffer by the NTW-array. (B) Bright-field image of the SCN slice after staining with TB. Scale bars, 100 µm.


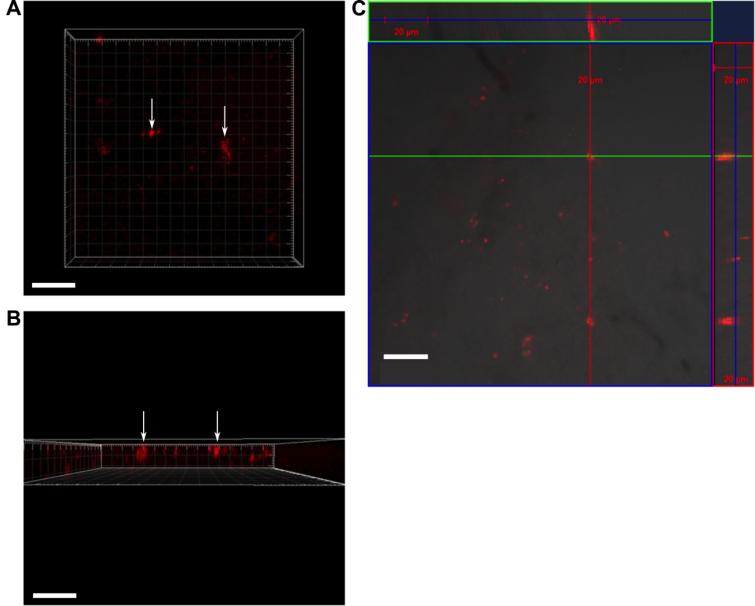


**Figure S3. Red FP (mCherry) transgene injected into the SCN slice.**

(A, B) Three-dimensional image of the SCN slice with the mCherry red signal following injection using an NTW array with a 25-µm wire length. White arrows show cells containing mCherry plasmid DNA within the SCN slice. Scale bars, 30 µm. (C) Two-dimensional imaging (*x*-*y*, *x*-*z*, *y*-*z* axial surface) of the mCherry signal in (B). Scale bar, 20 µm.

**Figure S4. Venus transgenes injected into an SCN slice of wild-type mice using chip lacking NTWs.**

(A) BF image and Venus of the SCN slice injected Venus plasmid DNA by chip without NTWs are shown as negative controls. Images using 10× magnification lens. Scale bar: 100 µm. (B) With the SCN slice, negative controls (injected Venus DNA by chip without NTWs) were lower than two types of positive controls (injected Venus DNA by a chip with NTWs). The SCN slice as the positive control injected Venus DNA using a chip, with NTWs derived from wild-type and *Per1::luc* Tg mice (SD, n = 5). Significant differences are depicted as *p* < 10^−7^ *** using Dunnett's post hoc test among positive control samples to negative control samples of the injected Venus DNA using a chip lacking NTWs (R software).

**
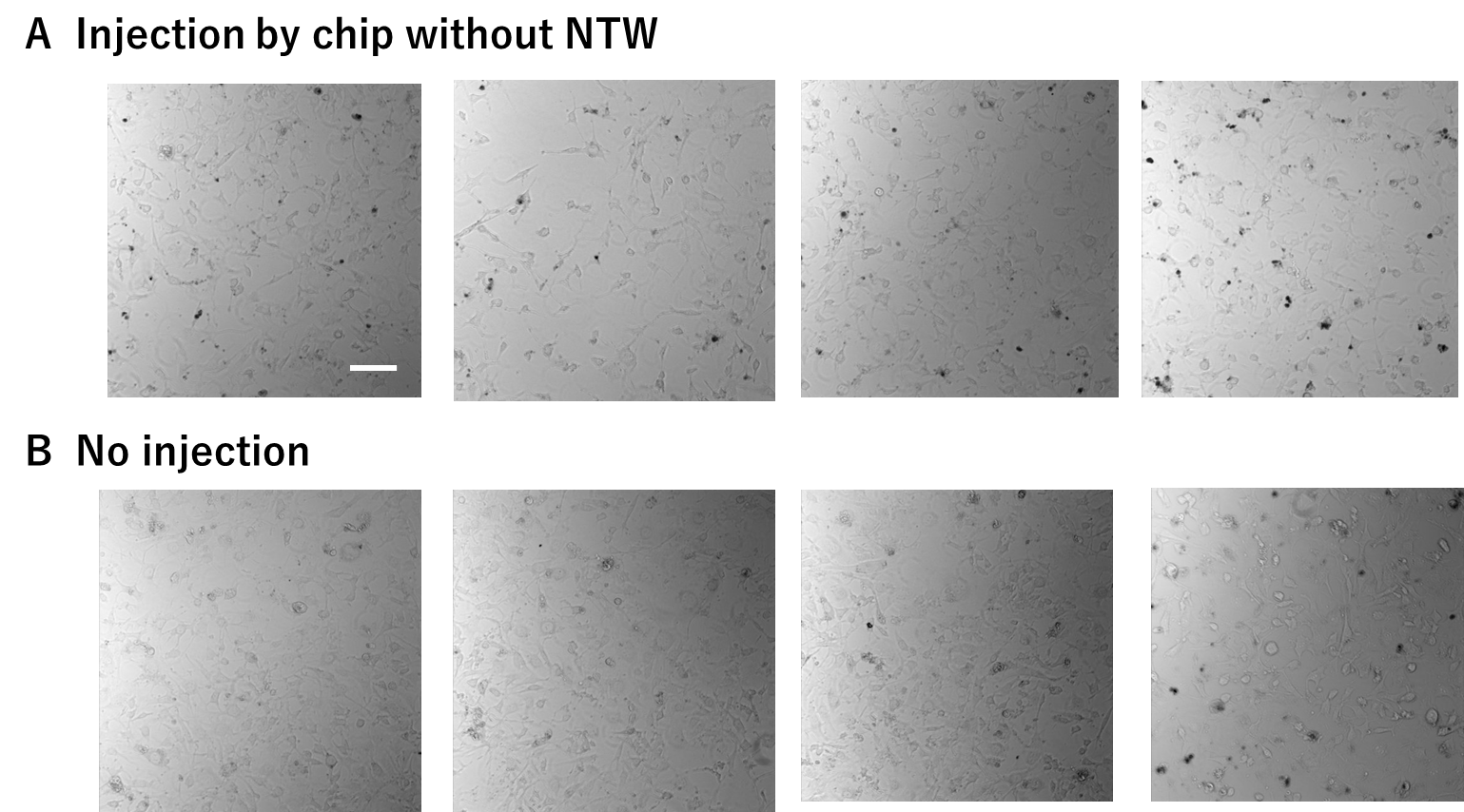
**

**Figure S5. Fibroblast cells dyed using** **TB after injection using chip without NTW or without injection itself.**

The same chip without NTW was placed at a 200 µm depth using a manipulator on the surface of cells on the half side bottom of the culture dish, while cells were not injected on another half at the bottom of the culture dish. (A) Images at the upper line indicate fibroblast cells injected by chip without NTW after staining with TB. (B) Images in the upper line show fibroblast cells without injection after staining with TB. Scale bar: 100 µm.


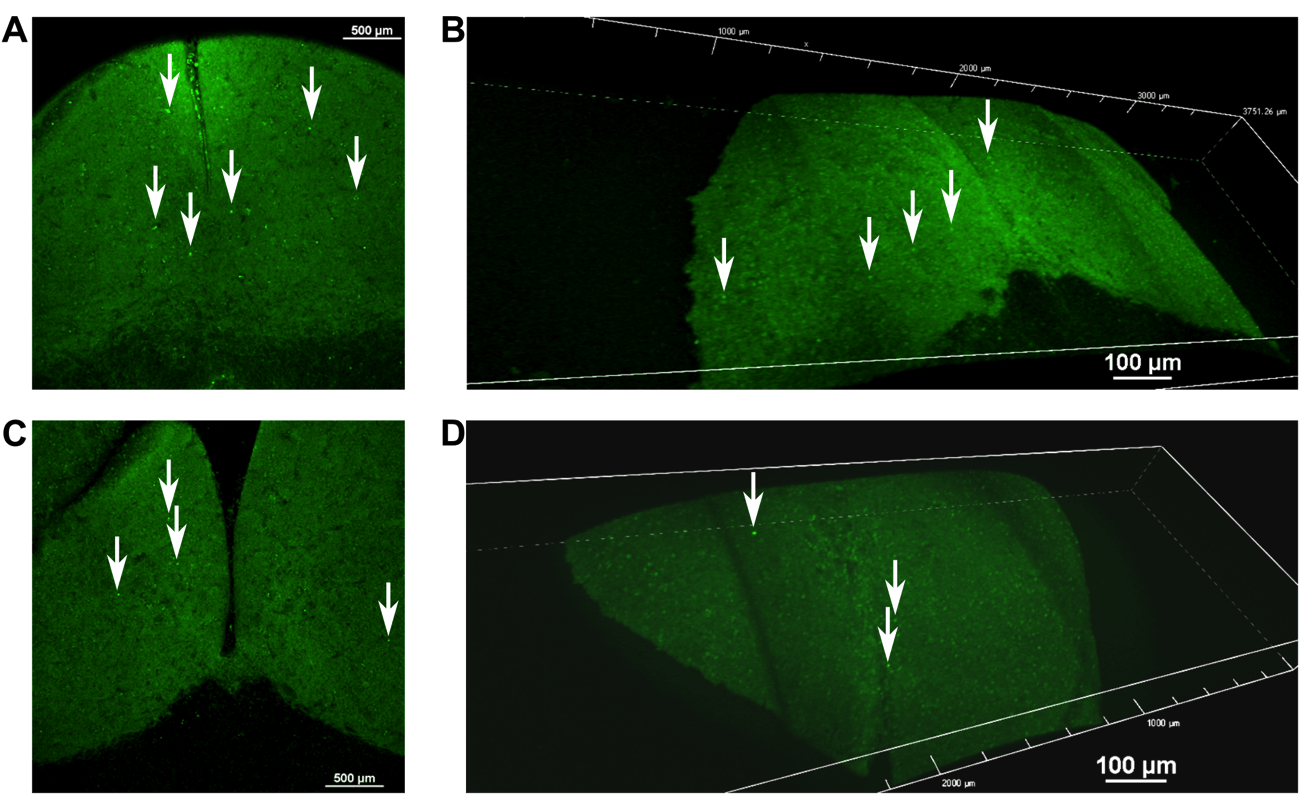


**Figure S6. Genome editing of cells in the SCN slice by CRISPR-Cas9 with an NTW array.**

The GFP protein is expressed by cutting an interrupted fragment DNA off the CRISPR-Cas9 system [pX330-Cetn1/1, pCAG-EGxxFP-Cetn1(Addgene)]. (A, B) Several cells with GFP signal were observed in the SCN slice upon a two-day incubation after injecting four times with CRISPR-Cas9 DNA by a 100-µm NTW array in 2D and 3D. (C, D) Similar 100-µm NTW arrays injected several cells with GFP signals in the SCN slice, 2D and 3D.


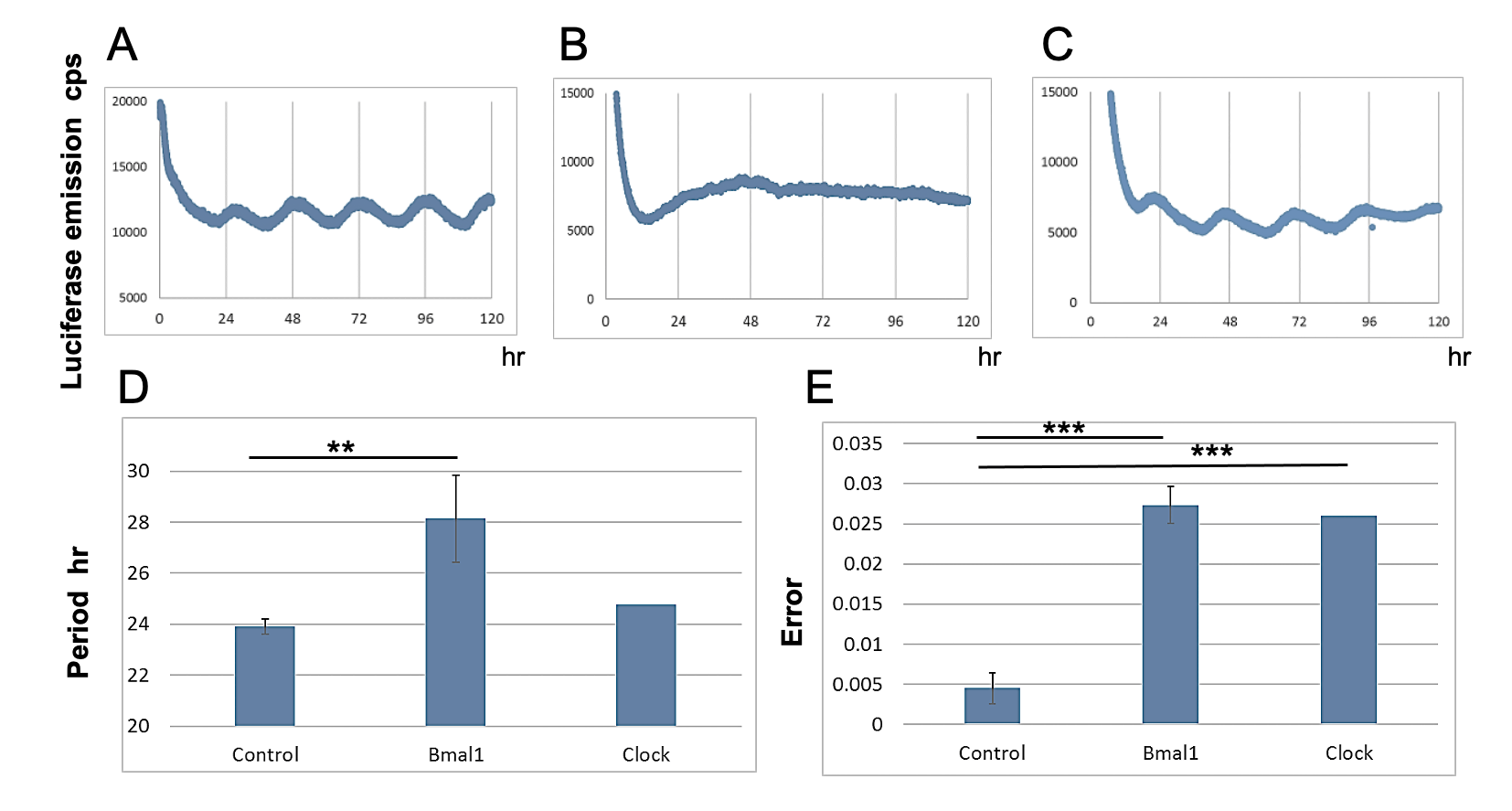


**Figure S7. Luciferase emission rhythms of the SCN slice after NTW-array RNAi injection.**

(A) Luciferase emission rhythms observed after injecting only buffer into the SCN slice of *Per1::luc* Tg. mice. (B) Luciferase emission rhythms observed after injecting *Bmal1* RNAi into the SCN slice of *Per1::luc* Tg. Mice. (C) Luciferase emission rhythms observed after injecting *Clock* RNAi into the SCN slice of *Per1::luc* Tg. mice. A software (Kai-Seki Ninja SL00-01; Churitsu Electric Corporation, Nagoya, Aichi, Japan) was used for these analyses (A–C). (D, E) *Per1*::*luc* emission rhythms of *Per1::luc* Tg SCN were measured, following *Bmal1*, *Clock* shRNA, or only buffer injection by NTW-array. The period length and error (differently from cosinor fitting) of missional rhythms were 28.1 h and 0.0273 after injecting *Bmal1* shRNA knockdown by software analysis (Ninjya SL00-01, Churitsu, Japan). The period length and the error of *Per1::luc* emission rhythms were 23.9 h and 0.024 after injecting *Clock* shRNA knockdown compared with 23.9 h and 0.0036 without shRNA injection and only buffer injection (control). Cosinor-fitting analyses of the luciferase emission rhythms of the SCN slice by NINJYA. D: period length, E: Cosinor curve fitting error of luciferase emission rhythms of the SCN slice. Figure S7D’s graph shows the period of emission rhythms in *Per1::luc* Tg mice based on Figure S8A-C data. The SCN slice period when injecting *Bmal1* shRNA was longer with repressed rhythms than injecting *Clock* shRNA (SD, n = 3). Figure S8E's graph shows the cosinor-fitting error of emission rhythms in *Per1::luc* Tg mice from Figure S8A-C data. The cosinor-fitting error of the SCN slice when injecting *Bmal1* shRNA was more significant with repressed rhythms than when injecting *Clock* shRNA (SD, n = 3). Dunnett's test was used to compare rhythmicity differences between knockdown groups by RNAi and control groups. Statistical significance was set at **:*p* < 0.001; ***: *p* < 0.0001.


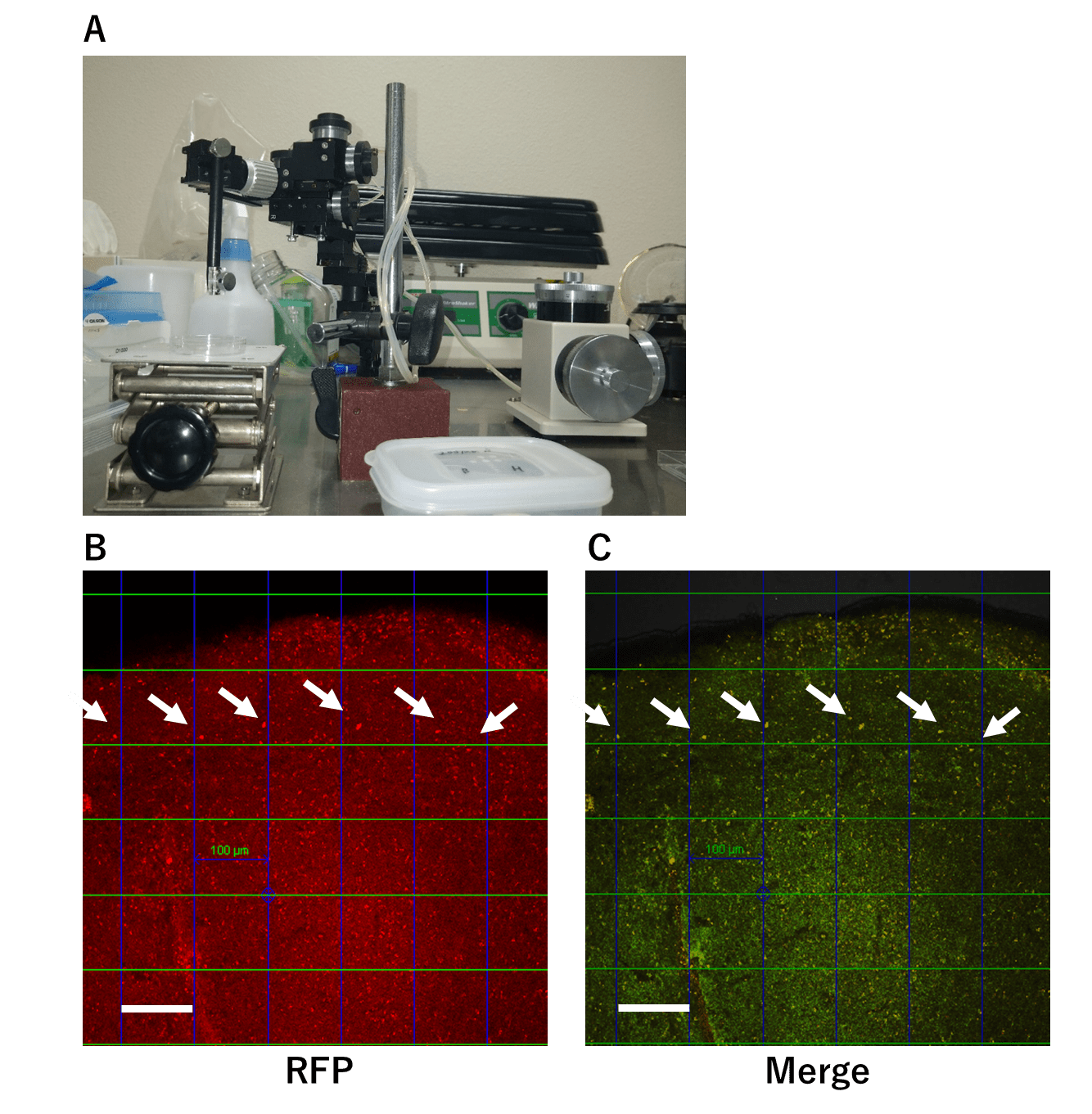


**Figure S8. RFP plasmid DNA was injected into the SCN slice of *Per1::*GFP Tg mice by a single NTW.**

(A) Injection system into the SCN slice using a single probe of NTW. The probe's position can be moved using a manipulator in x, y, z-axis directions. The probe was inserted into the SCN slice (300 µm thick on an agarose gel) to a 400 µm depth, after which the 400 ng/µL DNA solution was scattered over the SCN slice. (B) RFP image of the SCN slice from *Per1::*GFP Tg mice cultured one day after injecting RFP expression plasmid DNA. Six cells with red fluorescence signal (white arrows) were aligned with 100 µm pitch after injecting the RFP plasmid DNA using a single probe NTW with the manipulator. (C) Merged images between GFP and RFP in the injected SCN slice of *Per1::*GFP Tg mice showing six cells with both green and red fluorescence signals. In neuronal cells, the GFP was strongly expressed during the daytime, associated with the high transcription level of *Per1.* Scale bar, 100 µm.

**
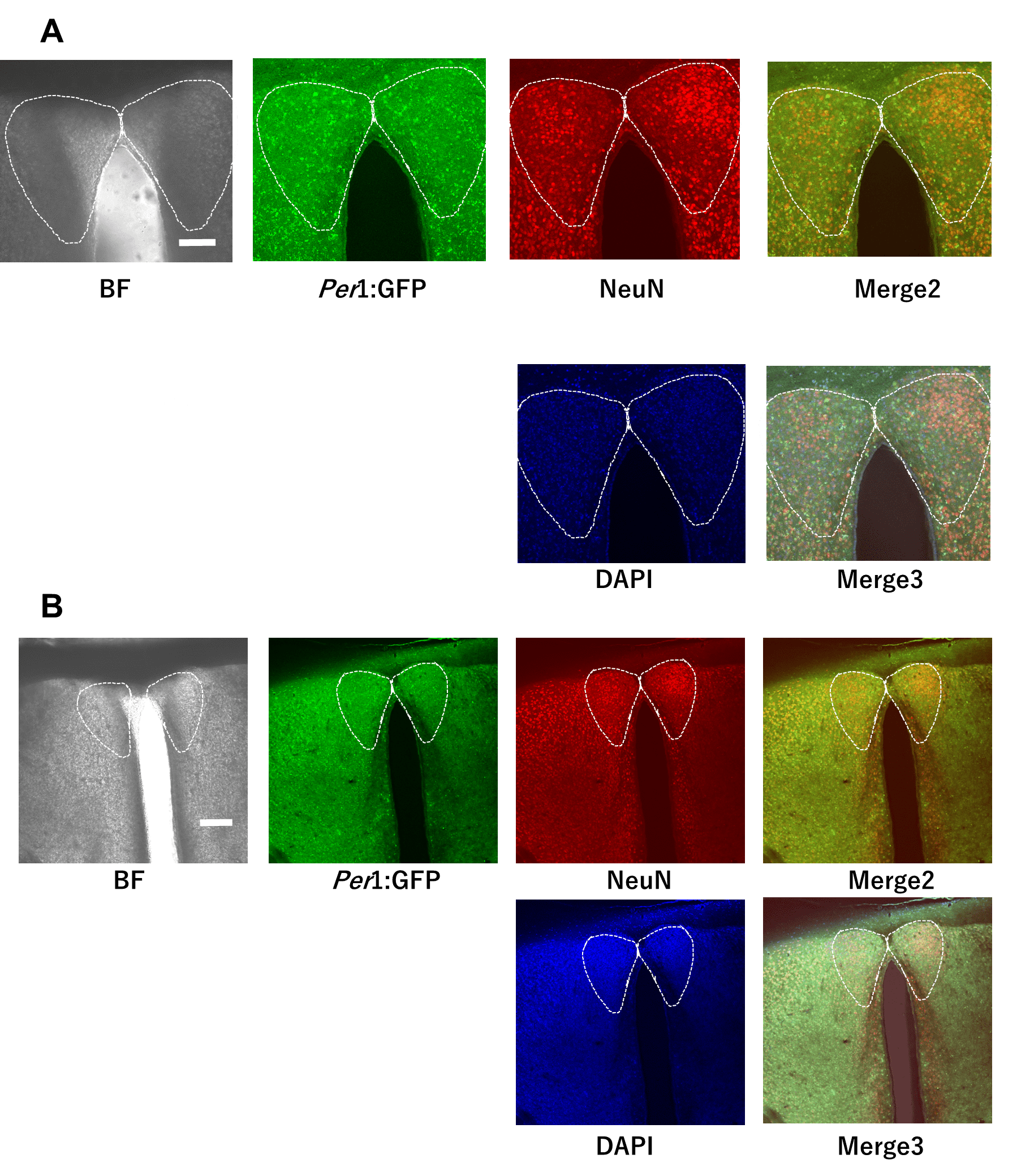
**

**Figure S9. Immunohistochemical images showing an SCN slice in *Per1::*GFP Tg mice at ZT8**.

BF image, *Per1::*GFP, and NeuN show images of the SCN slice in *Per1::*GFP Tg mice at ZT8 when Tg mice were bred in 12:12 h light: dark condition, eight hours after light-on time in daytime. (A) *Per1's* expression can be estimated from the GFP signal of a *Per1::*GFP image, and NeuN's expression in neuronal-specific cells can be assessed using Alexa Fluor 647's signal with a 20x magnification lens. Scale bar, 50 µm. (B) Using a 10x magnification lens. Scale bar, 100 µm. Merge1 images between BF, GFP, and Alexa Fluor 647 images. Merge2 images between BF, GFP, Alexa Fluor 647, and DAPI images. The SCN's was within the white line on the brain slice.

**
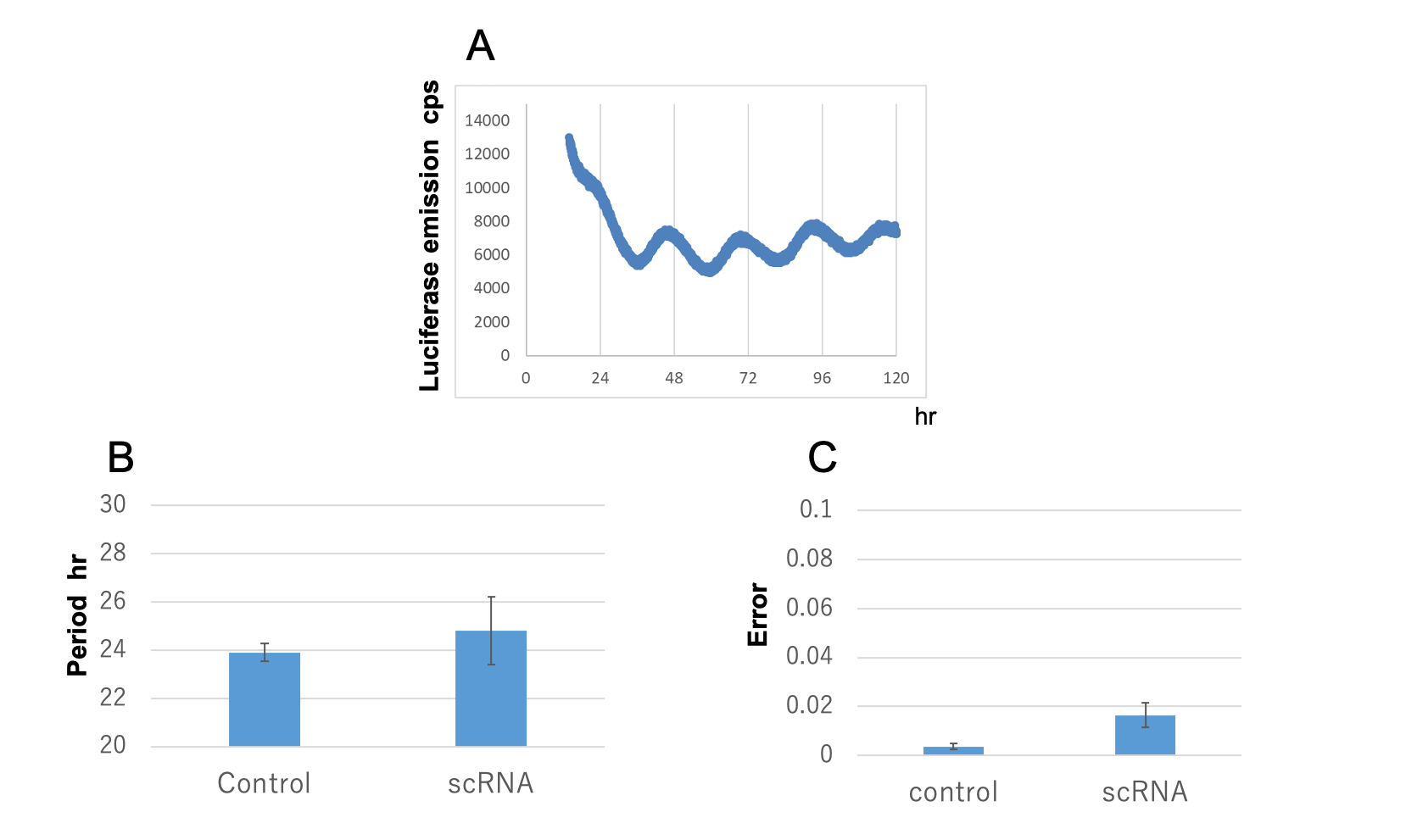
**

**Figure S10 Luciferase emission rhythms obtained from the SCN slice after NTW-array scRNA injection.**

(A) Luciferase emission rhythms observed after injecting scRNA into *Per1::luc* Tg SCNs by an NTW array. (B, C) The period length and error (differently from cosinor fitting) of emission rhythms were 24.8 h and 0.017 with scRNA injection compared with 23.9 h and 0.0036 without shRNA injection (control: PBS buffer only) (SD, n = 3). With an unpaired Student's t-test in the period length (*p* = 0.9202), neither the PBS buffer control nor the scRNA control was assessed for statistical significance. In error, both controls using the PBS buffer and scRNA were also unassessed by the Welch two Sample t-test. (*p* = 0.0059).
